# Overexpression of *MsASMT1* Promotes Plant Growth and Decreases Flavonoids Biosynthesis in Transgenic Alfalfa (*Medicago sativa* L.)

**DOI:** 10.3389/fpls.2020.00489

**Published:** 2020-04-28

**Authors:** Huifang Cen, Tingting Wang, Huayue Liu, Hui Wang, Danyang Tian, Xue Li, Xin Cui, Cong Guan, Hui Zang, Mengqi Li, Yunwei Zhang

**Affiliations:** College of Grassland Science and Technology, China Agricultural University, Beijing, China

**Keywords:** transgenic, *MsASMT1*, melatonin, flavonoids, lignin, alfalfa

## Abstract

Melatonin (N-acetyl-5-methoxytryptamine) is a pleiotropic signaling molecule that plays important roles in plant growth, development and stress responses. Alfalfa (*Medicago sativa* L.) is an important and widely cultivated leguminous forage crop with high biomass yield and rich nutritional value. The effects of exogenous melatonin content regulation on alfalfa stress tolerance have been investigated in recent years. Here, we isolated and introduced the *MsASMT1* (N-acetylserotonin methyltransferase) gene into alfalfa, which significantly improved the endogenous melatonin content. Compared with wild-type (WT) plants, *MsASMT1* overexpression (*OE-MsASMT1*) plants exhibited a series of phenotypic changes, including vigorous growth, increased plant height, enlarged leaves and robust stems with increased cell sizes, cell numbers and vascular bundles, as well as more branches. We also found that the flavonoid content and lignin composition of syringyl to guaiacyl ratio (S/G) were decreased and the cellulose content was increased in *OE-MsASMT1* transgenic alfalfa. Further transcriptomic and metabolomic exploration revealed that a large group of genes in phenylalanine pathway related to flavonoids and lignin biosynthesis were significantly altered, accompanied by significantly reduced concentrations of the glycosides of quercetin, kaempferol, formononetin and biochanin in *OE-MsASMT1* transgenic alfalfa. Our study first uncovers the effects of endogenous melatonin on alfalfa growth and metabolism. This report provides insights into the regulation effects of melatonin on plant growth and phenylalanine metabolism, especially flavonoids and lignin biosynthesis.

## Introduction

Melatonin (N-acetyl-5-methoxytryptamine) is a ubiquitous molecule in higher plants and is broadly known as a biological regulator of circadian rhythm and plant growth, development, senescence and stress responses (Reiter et al., 2015; Wang et al., 2018). Melatonin synthesis occurs in chloroplasts and mitochondria (Tan et al., 2013), which has been determined in various species; however, melatonin levels vary dramatically among different plant species and organs (Reiter et al., 2007).

The biosynthesis of melatonin in plants derives from the essential amino acid tryptophan, which is catalyzed successively by tryptophan decarboxylase (TDC), tryptamine 5-hydroxylase (T5H), serotonin N-acetyltransferase (SNAT) and N-acetylserotonin methyltransferase (ASMT)/caffeic acid O-methyltransferase (COMT). ASMT catalyzes the ultimate melatonin biosynthetic step and plays a vital role in melatonin biosynthesis in plants (Byeon et al., 2014; Tan et al., 2016).

With the sessile nature of plants, they are constantly challenged by various biotic and abiotic stresses during growth and development. Plants have evolved a series of defense mechanisms to protect them from adverse conditions. Melatonin is well known as a free radical scavenger and potent antioxidant. The endogenous or exogenous regulation of melatonin significantly alleviates oxidative damage in plants under drought (Antoniou et al., 2017), salinity (Liang et al., 2015), cold (Fu et al., 2017), heat (Shi et al., 2015; Xu et al., 2016), high light (Lee and Back, 2018a), and pathogen/pest stresses (Mandal et al., 2018; Wei et al., 2018). Melatonin is also essential for plant growth and development, such as seed germination (Posmyk et al., 2009; Zhang et al., 2014), lateral root formation (Park and Back, 2012), flowering (Byeon and Back, 2014; Zhang et al., 2018) and senescence (Lee and Back, 2017). The functional diversification of melatonin in plants is accompanied by crosstalk with various plant hormones, such as auxin, ethylene (ET), abscisic acid (ABA), salicylic acid (SA), jasmonic acid (JA), brassinosteroid (BR), and strigolactone (SL) (Arnao and Hernández-Ruiz, 2018a, 2019; Lee and Back, 2018b; Zhang Z. et al., 2019).

Melatonin also functions in metabolic processes, including primary metabolism (Zhao et al., 2015; Kobyliñska et al., 2018) and secondary metabolism (Yuan et al., 2016; Zhang et al., 2016; Ai and Zhu, 2018). Pretreatment with melatonin improved the accumulation of anthocyanin by upregulating the transcript levels of anthocyanin biosynthetic genes in cabbage (Zhang et al., 2016). However, melatonin antagonized JA in anthocyanin biosynthesis in *Arabidopsis* (Ai and Zhu, 2018). Our previous research found that genes related to flavonoid biosynthesis and phenylalanine metabolism pathways were upregulated in ovine *HIOMT* overexpression switchgrass plants (Yuan et al., 2016), which suggests that melatonin may participate in the biological process of flavonoid biosynthesis. Flavonoids are widely distributed throughout the plant kingdom, functioning in plant growth and development and in responses to various biotic and abiotic stresses, including UV protection and defense responses to pathogens/insects (Kumar and Pandey, 2013; Mitsunami et al., 2014). Lee et al. (2018) found that flavonoids acted as potent inhibitors of melatonin synthesis. The exogenous application of flavonoids (morin and myricetin) or the overexpression of a putative flavonol synthase gene (*FLS*) resulted in the increased production of flavonoids but reduced accumulation of melatonin in rice. However, it is still unclear whether high levels of melatonin affect flavonoids biosynthesis, and which flavonoid are mainly regulated.

In human beings, melatonin is widely known as a dietary supplement that improves the quality of sleep (Cornu et al., 2010), has anti-aging properties (Tresguerres et al., 2014) and alleviates the effects of jet lag (Waterhouse et al., 1997). Numerous pharmacological studies confirmed that melatonin could also be associated with the prevention of several diseases, including cardiovascular and neurodegenerative diseases or cancer (Claustrat et al., 2005; Arnao and Hernández-Ruiz, 2018b). Alfalfa (*Medicago sativa* L.) is an important and widely cultivated leguminous forage crop worldwide and is known as the “King of Forages,” mainly due to its high biomass yield, high protein content and rich nutritional value (Aung et al., 2015; Fu et al., 2015). Alfalfa has been successfully used as a raw material for health products, and its dietary market is developing rapidly. Improving the melatonin content in alfalfa has profound implications for improving forage quality and nutritional value. To date, there are only two reports related to the function of melatonin in alfalfa on improving drought (Antoniou et al., 2017) and waterlogging resistance (Zhang Q. et al., 2019) with exogenous melatonin pretreatment, while the function of endogenous melatonin in alfalfa has not been reported. Alfalfa is rich in various flavonoids, including quercetin, luteolin, formononetin, and so on. It is of great significance to explore the effect of melatonin on flavonoids biosynthesis in alfalfa.

Here, we report that the overexpression of *MsASMT1* in alfalfa led to melatonin accumulation but exhibited a contrasting effect on flavonoids accumulation. In this report, we first cloned the N-acetylserotonin methyltransferase (*ASMT*) gene from alfalfa and produced *OE*-*MsASMT1* transgenic alfalfa by *Agrobacterium*-mediated transformation. The growth and development phenotype of *OE-MsASMT1* transgenic alfalfa were evaluated compared to WT plants. *OE-MsASMT1* transgenic alfalfa had a higher melatonin content and lower flavonoid content. Further transcriptomic and metabolomic analyses found a large group of genes in phenylalanine pathway were downregulated and the concentration of quercetin, kaempferol, formononetin, and biochanin were decreased. Our study first reported the effects of endogenous melatonin on alfalfa plant growth and indicated that melatonin biosynthesis inhibited flavonoids accumulation in alfalfa plants.

## Materials and Methods

### Gene Isolation, Bioinformatics Analysis and Vector Construction

The apple *ASMT* and rice *ASMT1* genes were obtained for BLAST queries against *Medicago truncatula* resources at https://phytozome.jgi.doe.gov/, and *MtASMT* (*Medtr5g074600.1*) was found to share high identity (60.20%) with apple *ASMT* and relatively lower identity (38.33%) with rice *ASMT1*. Due to homology and phylogenetic analysis between alfalfa and *M. truncatula*, primers were designed based on the *M. truncatula ASMT* sequence to amplify the coding sequence (CDS) in the alfalfa genome.

Total RNA was extracted from the leaves, stems and roots of alfalfa using TRIzol reagent (Invitrogen, Carlsbad, CA, United States), and intact RNA was immediately reverse transcribed using the PrimeScript RT Reagent Kit with gDNA Eraser (Takara, Dalian, China) according to the manufacturer’s instructions. cDNAs were used as templates to amplify the CDS of MsASMT1 (GenBank accession number: MN092350); primers are listed in Supplementary Table S1.

PCR products were verified by sequencing. Nucleotide sequence and amino acid sequence alignments were performed with DNAMAN. The conserved domain^1^ and physicochemical properties^2^ of MsASMT1 were analyzed online.

Amino acid sequences of alfalfa (*M. sativa*), *M. truncatula* and other selected species (*Oryza sativa* OsASMT1 (AK072740), OsASMT2 (AK069308), OsASMT3 (AAL34945.1), *Malus zumi* MzASMT (KJ123721), *Phaseolus vulgaris* PvASMT (XP_007142077), *Setaria italica* SiASMT (XP_004973630.1), *Triticum urartu* TuASMT (EMS45259.1), *Brachypodium distachyon* BdASMT (XP_003571634.3), *Cicer arietinum* CaASMT (XM_004490639.2), *Cucumis sativus* CsASMT (XP_004151735), *Glycine max* GmASMT (XP_003536188), *Hordeum vulgare* HvASMT (BAK00281), and *Panicum hallii* PhASMT (XP_025822442.1)] were aligned using Clustal X 1.83, and a neighbor-joining phylogenetic tree was constructed based on the amino acid sequence using MEGA 7 software (Park et al., 2013; Zuo et al., 2014; Byeon et al., 2015).

To construct the overexpression vector, the *MsASMT1* complementary DNA fragment was amplified using unique primers containing restriction sites for *Bam*HI/*Kpn*I and cloned into the destination vector pZh01. The pZh01*-MsASMT1* vector was introduced into *Agrobacterium* strain EHA105.

### Subcellular Localization of *MsASMT1*

For subcellular location, MsASMT1 was fused to the C terminus of GFP in *pBWA(V)HS-GLosgfp* by *Eco*RV. One-month-old *Nicotiana benthamiana* leaves were used for infiltration with the GV3101 strain carrying pBWA(V)HS-MsASMT1-GLosgfp. The subcellular localization of MsASMT1 was determined using a Nikon C2-ER laser confocal microscope after two days.

### Circadian Rhythm Analysis of *MsASMT1* Gene and Melatonin Content

For circadian rhythm analysis of *MsASMT1* gene, total RNA was extracted from the leaves of wild type (WT) plants at different time points (3:00, 6:00, 9:00, 12:00, 15:00, 18:00, 21:00, and 24:00) during one day. cDNAs were generated by reverse transcription enzyme, and the relative expression level of *MsASMT1* gene were analyzed by quantitative real-time PCR (qRT-PCR) with the specific primer (MsASMT1-F, MsASMT1-R).

For circadian rhythm analysis of melatonin, the melatonin contents of WT plant leaves at the same time points with *MsASMT1* gene expression analysis were extracted and quantified using a plant melatonin enzyme-linked immunosorbent assay (ELISA) kit (Suzhou Comin Biotechnology Co., Ltd., Suzhou, China) according to the manufacturer’s protocol.

### *Agrobacterium*-Mediated Transformation

*Agrobacterium* strain EHA105 harboring pZh01*-MsASMT1* was transformed into alfalfa (*Medicago sativa* L.) cultivar ‘Zhongmu No. 1.’ The T-DNA region of pZh01*-MsASMT1* is illustrated in Supplementary Figure S1A. A modified *Agrobacterium*-mediated transformation protocol was used in this paper (Zhang and Wang, 2015). Cotyledons and hypocotyls from the seven-day-old seedlings were used as explants for transformation. After *Agrobacterium* inoculation, explants were subjected to cocultivation for 3 days and then transferred to callus induction medium for one month with 5 mg/L hygromycin B (hyg). The induced calluses were transferred to differential medium for somatic embryo induction, and the medium was changed every 2 weeks until torpedo-shaped embryos emerged. Then, the green buds were transferred to MS medium for further germination and plantlet formation. The resistant plantlets were transplanted to flowerpots filled with a mixture of vermiculite and humus, and the plants were maintained in a greenhouse under a 16 h/8 h (light/dark) photoperiod with 200 μmol m^–2^s^–2^ light intensity at 25 ± 2°C.

### Transgenic Plants Verification

Total genomic DNA from every transgenic plant was extracted using the CTAB method. PCR was performed to confirm the positive transformants using specific primers of the selectable marker gene *hpt* for *MsASMT1* gene were derived from alfalfa plants and which could be detected in WT plants.

Total RNA was extracted from leaves of different *OE-MsASMT1* transformants. cDNAs were generated by reverse transcription enzyme. Positive transgenic plants were selected randomly for RT-PCR and qRT-PCR tests, which were performed with a pair of primers (MsASMT1-F, MsASMT1-R) specific to *MsASMT1* gene. An *actin* gene of alfalfa was used as the internal control for RNA normalization (Castonguay et al., 2015).

The relative expression levels of *MsTDC*, *MsT5H*, *MsSNAT*, and *MsCOMT* in positive transgenic plants were analyzed by quantitative real-time PCR with the specific primers listed in Supplementary Table S1.

### Quantification of the Melatonin Content

Melatonin was extracted with a modified method according to Zhang et al. (2014). Approximately 2 g of fresh leaves and stems from the top 10 cm portion were ground into powder with liquid nitrogen and then homogenized with 5 mL methanol at −20°C for 30 min. Homogenate was ultrasonicated at 45°C for 40 min. After centrifugation at 12000 rpm at 4°C for 15 min, the supernatants were collected and dried using nitrogen gas. The extracts were dissolved in 5% methanol and purified using a C18 solid phase extraction (SPE) column (Kromasil, 250 mm × 4.6 mm, 5 μm). The cartridge was then washed with 1 mL 5% methanol, and melatonin was finally eluted with 1 mL 80% methanol. The extract was subsequently filtered through a 0.22 μm PTFE syringe filter before UHPLC-ESI-MS/MS analysis. Melatonin determination and quantification was analyzed using a UHPLC-ESI-MS/MS (Rigol L3000, China).

### Phenotypic and Histochemical Analyses

The WT plants and *OE-MsASMT1* transgenic plants were cultivated in a greenhouse for two months. Five *OE-MsASMT1* transgenic plants (OE3, OE12, OE15, OE16, and OE25) were selected for the comparison of leaf size, stem diameter and plant height with several WT plants. The second fully expanded mature leaves from the top were selected for comparing leaf size. The stem diameter was measured 3 cm above the ground with four mature tillers of the same transgenic plants with a Vernier caliper.

The excised stems of the third internode from the top of three-month-old *OE-MsASMT1* transgenic alfalfa and WT plants were used for paraffin section analysis. Tissues were fixed in FAA (formalin-aceto-alcohol) solution for 24 h, dehydrated using 85% ethanol, permeated and embedded in wax. Cross sections were sliced for saffron and green staining to observe the cell morphology and vascular bundles.

### Quantification of the Lignin and Flavonoid Contents

Stems of the *OE-MsASMT1* transgenic plants (OE16, OE25) and WT plants were collected and dried at 65°C for 48 h for the lignin content measurement. The quantification of lignin content was performed according to Li et al. (2011) using a modified Klason method. Three biological replicates were performed.

Fresh samples from the top 10 cm portions of WT plants and *OE-MsASMT1* transgenic plants were harvested for total flavonoids content measurement. The extraction and quantification of total flavonoids were performed using a plant flavonoid kit (Suzhou Comin Biotechnology Co., Ltd) following the manufacturer’s instructions with a spectrophotometer (Hitachi UH5300, Japan). Three biological replicates were performed.

#### Quantification of IAA Content

Approximately 50 mg fresh samples from the top 10 cm portions of three-month-old WT plants and *OE-MsASMT1* transgenic plants (OE16, OE25) were ground into powder with liquid nitrogen. The samples were extracted in 0.5 mL extracting solution and 50 μL internal standard fluid at 4°C for 30 min. Then, the homogenate was extracted with 1 mL trichloromethane. After centrifugation at 14000 rpm at 4°C for 10 min, the subnatant was collected and dried using nitrogen gas. Extracts were dissolved in 100 μL methanol, filtered through a 0.1 μm membrane and transferred to sample vials for LC-MS analysis (UPLC I-Class; Q-Exactive MS). Three biological replicates were performed.

#### RNA-Sequencing

Fresh samples of the top 10 cm portions (leaves and stems) of three-month-old *OE-MsASMT1* transgenic plants (OE3, OE12, OE15, OE16, and OE25) and WT plants with five biological replications were used for RNA-seq analysis without a reference genome. The purity, concentration and integrity of the RNA samples were tested to ensure the use of qualified samples for RNA sequencing. A total of 1 μg RNA per sample was used as input material for the RNA sample preparations. The generation of a sequence library and gene functional annotation were performed in Biomarker Technology Co., Ltd. (Beijing, China). The raw data were submitted to the NCBI Sequence Read Archive (SRA) database^3^, and the BioProject accession number is PRJNA555673.

The RNA-seq results were validated by quantitative real-time PCR, the relative expression levels of 14 genes selected from the RNA-seq results were tested in WT plants and *OE-MsASMT1* transgenic plants (OE16 and OE25 with lower and moderate expression level of *MsASMT1* gene) with the specific primers listed in Supplementary Table S1.

#### Widely Targeted Metabolomic Analysis

The plant materials used for metabolomic analysis were the same as those used for RNA-seq analysis (OE3, OE12, OE15, OE16, OE25, and WT plants). Approximately 1.5 g of each fresh sample from *OE-MsASMT1* transgenic plants and WT plants were collected and frozen immediately in liquid nitrogen and stored at −80 °C. Then, the samples were delivered to Biomarker Technology Co., Ltd., for widely targeted metabolomic analysis.

#### Determination of Forage Quality

Shoots of *OE-MsASMT1* transgenic plants and WT plants at the squaring stage were harvested and dried at 65°C for 48 h. According to Reddy et al. (2005), dried samples were ground with a mill and passed through a 1 mm sieve. Approximately 50 mg dry powder samples were used for neutral detergent fiber (NDF), acid detergent fiber (ADF) and acid detergent lignin (ADL) analyses by a fiber analyzer (ANKOM 2000). Three biological replicates were performed.

#### Statistical Analysis

All experiments were performed with at least three biological replicates. The values presented are the means ± SE of three replications. Statistical analyses were conducted using one-way analysis of variance (ANOVA) with SPSS 18.0.

## Results

### Isolation of *MsASMT1* and Genetic Analysis

A full-length coding sequence (CDS) of 1077 bp was isolated from the alfalfa genome and shared up to 88.61% identity with *MtASMT* (*Medtr5g074600.1*) (Supplementary Figure S2A). This gene was named *MsASMT1*, and encodes a protein of 358 amino acids, contains a dimerization domain and is a S-adenosylmethionine-dependent methyltransferase (SAM or AdoMet-MTase superfamily), which is similar to *ASMT* genes reported in other plant species (Supplementary Figure S2B). The neighbor-joining phylogenetic tree clearly showed that the ASMTs clustered into two groups for monocots and dicots, which demonstrated that the *ASMT* gene was evolutionarily conserved (Supplementary Figure S3).

**FIGURE 1:**
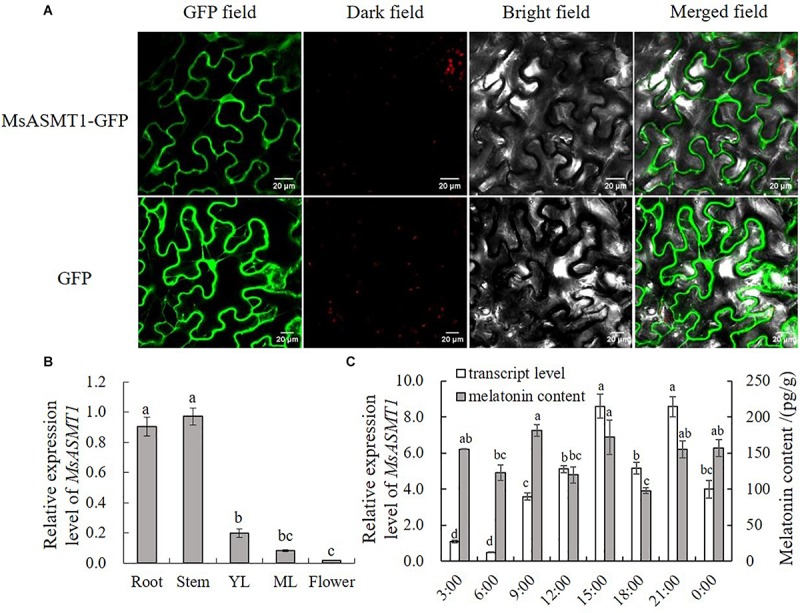
Characterization of *MsASMT1.* **(A)** Subcellular location of MsASMT1 in *Nicotiana tabacum.* From left to right: green fluorescence, dark field, bright field, merged field of MsASMT1-GFP and GFP; **(B)** Relative expression level of *MsASMT1* in different tissues of alfalfa. YL, young leaves, ML, mature leaves. The letters indicate the differences among different tissues of alfalfa according to ANOVA analysis (*p* < 0.05); **(C)** Circadian rhythm of *MsASMT1* transcript levels and melatonin content in alfalfa throughout one day. Data are presented as means ± SE (*n* = 3), the letters indicate the differences of *MsASMT1* transcript levels and melatonin content respectively among different time points according to ANOVA analysis (*p* < 0.05).

To further understand the characteristics of *MsASMT1*, we performed subcellular localization and determined the expression patterns of *MsASMT1* in different tissues by quantitative real-time PCR. The results showed that MsASMT1 was mainly located in cytoplasm, and also existed in nucleus (Figure 1A). As shown in Figure 1B, *MsASMT1* was mainly expressed in the stems and roots, followed by young and mature leaves, while the *MsASMT1* gene was rarely expressed in flowers, indicating that *MsASMT1* might especially function in stem and root growth. Because melatonin plays an important role in plant circadian rhythm, we investigated the melatonin levels and *MsASMT1* expression levels in alfalfa throughout one day. As shown in Figure 1C, the peak of *MsASMT1* expression occurred at 15:00 pm and 21:00 pm, while the melatonin content peaked at 9:00 am and 15:00 pm and remained at a stable level at night from 21:00 pm to 3:00 am.

### Overexpression of *MsASMT1* Increases Melatonin Biosynthesis in Transgenic Plants

**FIGURE 2:**
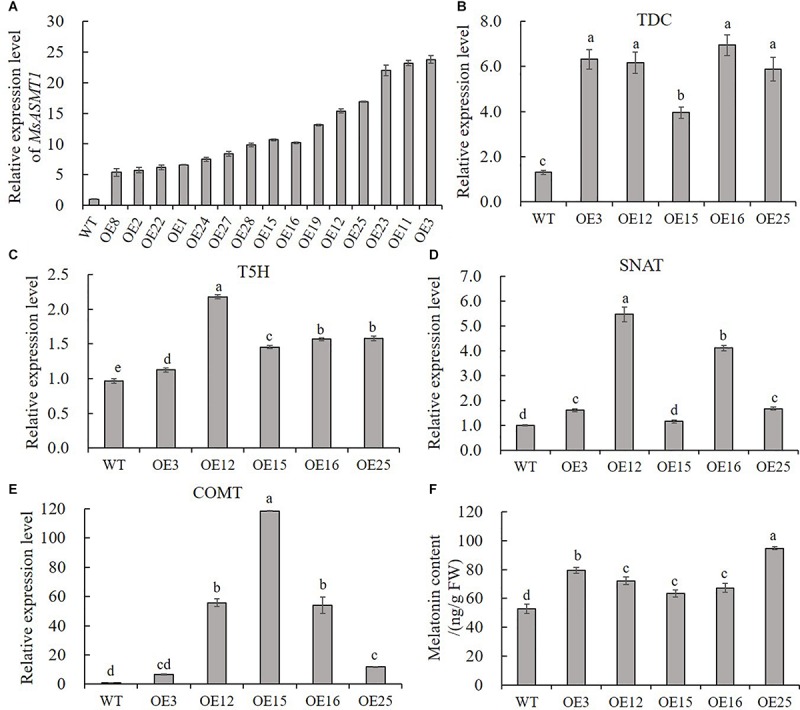
Relative expression level of *MsASMT1* and the upstream genes of *ASMT* in melatonin biosynthesis pathway and the melatonin content in WT plants and *OE-MsASMT1* transgenic plants. **(A)** Relative expression level of *MsASMT1* in various transgenic alfalfa plants; **(B–E)** *MsTDC* **(B)** *MsT5H* **(C)** *MsSNAT* **(D)** and *MsCOMT* **(E)** in WT plants and *OE-MsASMT1* transgenic plants; **(F)** Melatonin content in WT plants and *OE-MsASMT1* transgenic plants, the correlation between melatonin content and relative expression level of *MsASMT1* is analyzed by SPSS with Pearson Correlation Coefficient (*p* < 0.05). Data are presented as mean ± SE (*n* = 3). The letters indicate the differences between WT and *OE-MsASMT1* transgenic plants according to ANOVA analysis (*p* < 0.05).

To characterize the function of *MsASMT1* in alfalfa, we generated a large number of *MsASMT1*-overexpressing (*OE-MsASMT1*) transgenic plants using tissue culture (Supplementary Figures S1B–E). And most of them were PCR-positive, the expected size of the selectable marker gene *hpt* (741 bp) was detectable in *OE-MsASMT1* transgenic plants (Supplementary Figure S4A). To further detect the expression of *MsASMT1* in transgenic plants, we performed RT-PCR and qRT-PCR, respectively. The results showed that *MsASMT1* gene had expressed in most of the tested transgenic plants (Supplementary Figures S4B,C). And the relative expression levels of *MsASMT1* in transgenic plants varied from 5 to 20 times higher than that in WT plants (Figure 2A). Transgenic plants OE3, OE12 with high expression level, OE25 with middle expression level and OE15, OE16 with low expression level were selected for further research.

Because ASMT catalyzes the last critical enzymatic step in melatonin biosynthesis, we wondered whether feedback in this pathway occurred once the expression level of *MsASMT1* changed in alfalfa. We analyzed the transcripts of *MsTDC*, *MsT5H*, *MsSNAT*, and *MsCOMT* in these transgenic plants. Quantitative real-time PCR analysis showed that the expression levels of all these genes in *OE-MsASMT1* transgenic plants were increased compared to those in WT plants (Figures 2B–E). These results indicated that the overexpression of *MsASMT1* directly or indirectly upregulated the expression of upstream genes.

Since ASMT is responsible for melatonin biosynthesis and with the upregulation of other genes in melatonin biosynthesis pathway, we directly measured the melatonin levels in the WT plants and *OE-MsASMT1* transgenic plants. The results revealed that the increases of melatonin content in *OE-MsASMT1* transgenic plants varied from 21.2% to 80.8% compared to that in WT plants (Figure 2F), and there was a positive correlation between melatonin content and the relative expression level of *MsASMT1*, the Pearson Correlation Coefficient is 0.782 without significant correlation.

### Overexpression of *MsASMT1* Increases Leaf and Stem Sizes and Increases Plant Growth in Transgenic Plants

**FIGURE 3:**
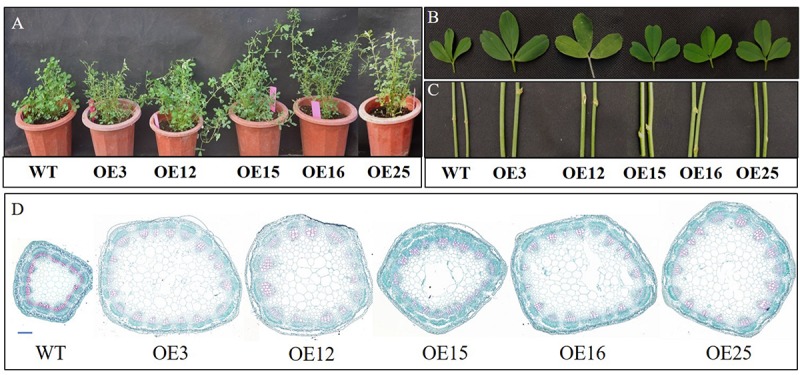
Effects of *MsASMT1* overexpression on alfalfa growth and development. **(A)** Growth situation of WT plants and *OE-MsASMT1* transgenic plants after growing in greenhouse for two months; **(B,C)** the leaf **(B)** and stem **(C)** comparison between WT plants and several *OE-MsASMT1* transgenic plants, scale bar, 1 cm; **(D)** Stem cross sections with safranin green staining of WT plants and *OE-MsASMT1* transgenic plants, scale bar, 1 mm.

Compared to WT plants, *OE-MsASMT1* transgenic plants exhibited vigorous growth with larger leaves and thicker stems (Figures 3A–C). The plant height of *OE-MsASMT1* transgenic plants ranged from 69.63 cm to 90.60 cm, which was significantly higher than that of WT plants (58.25 cm). The stem diameter of *OE-MsASMT1* transgenic plants ranged from 2.71 mm to 3.03 mm, which was significantly thicker than that of WT plants (1.97 mm; Table 1).

**TABLE 1:** Stem diameter and plant height of WT plants and *OE-MsASMT1* transgenic plants.

**Lines**	**Stem diameter/mm**	**Plant height/cm**
WT	1.97 ± 0.08b	58.25 ± 2.96d
OE3	2.84 ± 0.18a	75.75 ± 1.89bc
OE12	2.89 ± 0.07a	69.63 ± 1.25bc
OE15	2.71 ± 0.07a	74.25 ± 3.28bc
OE16	3.03 ± 0.10a	90.60 ± 4.83a
OE25	2.84 ± 0.05a	73.00 ± 0.91bc

*Each value represents the mean ± SE from at least three replicates. The letter following the value in the same row indicates the differences between WT plants and *OE-MsASMT1* transgenic plants according to ANOVA analysis (*p* < 0.05).*

In agreement with the morphological changes, histological analysis showed that the cell size, cell number and vascular bundle in *OE-MsASMT1* transgenic plant stems significantly increased compared to those in WT plants, the cells were disordered with irregular shapes and the intercellular spaces became more sparsely arranged (Figure 3D, Table 2).

**TABLE 2:** Stem microstructure indexes of WT plants and *OE-MsASMT1* transgenic plants.

**Lines**	**Cell numbers**	**Vessel**	**Cell perimeter/μm**	**Cell area/μm^2^**
WT	143.67 ± 8.45c	13.33 ± 0.88c	265.77 ± 14.62c	4335.30 ± 872.60e
OE3	269.33 ± 6.39a	20.00 ± 1.15a	550.17 ± 8.24a	18424.20 ± 458.40a
OE12	258.67 ± 9.05a	19.67 ± 0.95a	501.64 ± 7.13a	14315.24 ± 538.10c
OE15	193.33 ± 10.65b	16.33 ± 0.88bc	292.80 ± 15.46c	11417.45 ± 435.15d
OE16	243.33 ± 17.07a	18.33 ± 0.88ab	453.38 ± 10.20b	13948.57 ± 849.08c
OE25	262.00 ± 11.79a	18.00 ± 1.15ab	469.68 ± 16.53b	15740.03 ± 857.17b

*Each value represents the mean ± SE from three replicates. The letter following the value in the same row indicates the differences between WT plants and *OE-MsASMT1* transgenic plants according to ANOVA analysis (*p* < 0.05).*

**FIGURE 4:**
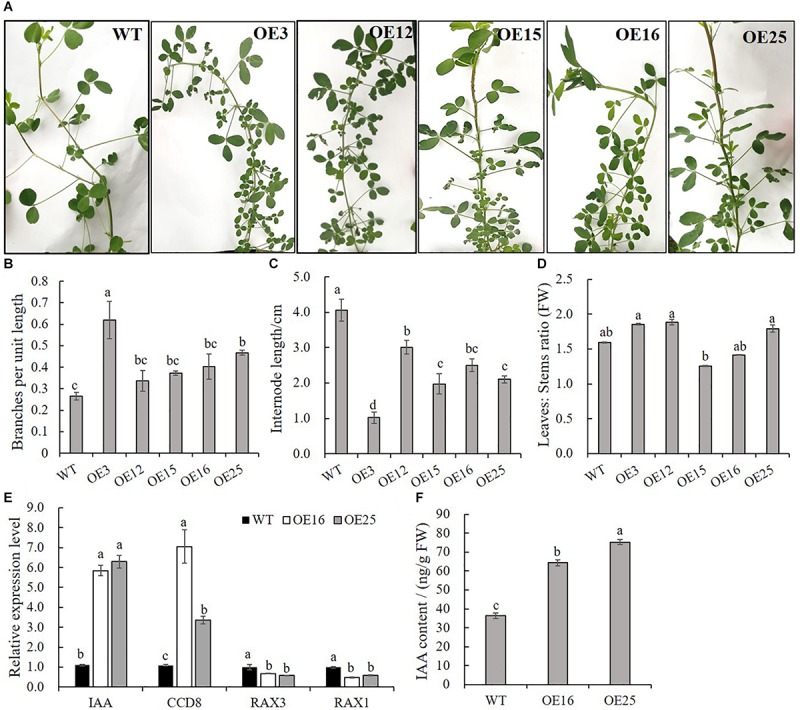
Branching characteristics of WT plants and *OE-MsASMT1* transgenic plants. **(A)** Branching phenotype of three-month-old WT plants and several *OE-MsASMT1* transgenic plants; **(B–D)** The number of lateral branches per unit length on the main branch **(B)**, the internode length between two lateral branches **(C)** and the ratio of fresh leaves to fresh stems **(D)** of WT plants and *OE-MsASMT1* transgenic plants after grown in greenhouse for three months; **(E)** Relative expression level of genes related to plant branching in WT plants and *OE-MsASMT1* transgenic plants; **(F)** IAA content of WT plants and *OE-MsASMT1* transgenic plants. Data are presented as mean ± SE (*n* = 3). The letters indicate the differences between WT and *OE-MsASMT1* transgenic plants according to ANOVA analysis (*p* < 0.05).

In contrast to WT plants, *OE-MsASMT1* transgenic plants exhibited bushy branches at the preliminary vegetative stage (Figure 4A). The number of branches per unit length of OE3 exhibited a 2-fold increase, and the internode length of OE3 was 75% shorter than that of WT plants (Figures 4B,C). The leaf/stem ratios of OE3, OE12, and OE25 were also increased compared with that of WT plants (Figure 4D). Identified genes that are known to be involved in branch formation, such as *IAA* (indoleacetic acid) and *CCD8* (carotenoid cleavage dioxygenase 8), were significantly increased in OE16 and OE25, while *RAX1* (regulator of axillary meristems 1) and *RAX3* exhibited a contrasting trend (Figure 4E) (Foo et al., 2005; Rubio-Moraga et al., 2014; Guo et al., 2015); the IAA content was also significantly increased in the top of *OE-MsASMT1* transgenic plants stems (Figure 4F).

### Overexpression of *MsASMT1* Reduces Flavonoids Biosynthesis and Changes Lignin Composition in Transgenic Plants

**FIGURE 5:**
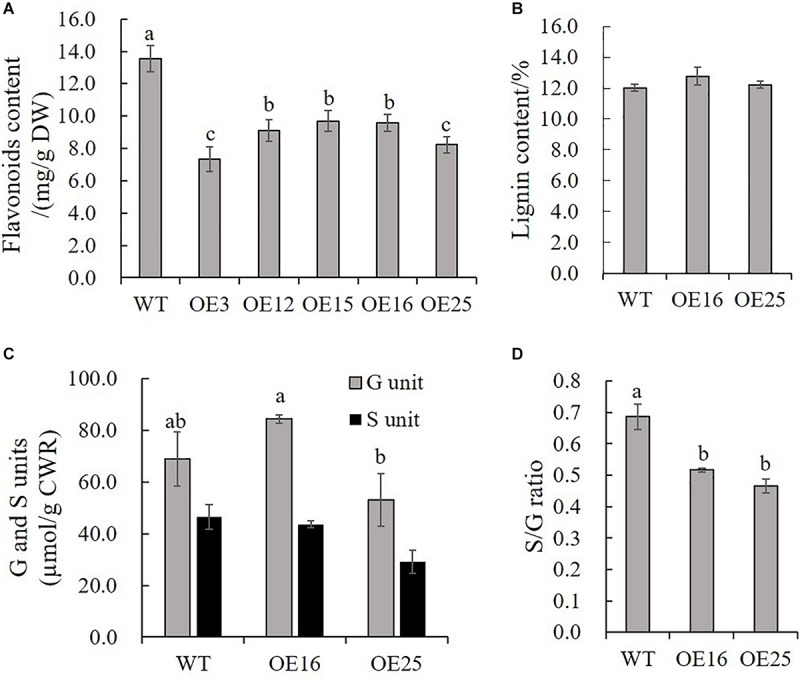
Flavonoids content and lignin content of WT plants and *OE-MsASMT1* transgenic plants. **(A,B)** Total flavonoids content **(A)** and lignin content **(B)** in WT plants and *OE-MsASMT1* transgenic plants; **(C)** The lignin monomer G and S unit content in WT plants and *OE-MsASMT1* transgenic plants; **(D)** The S/G ratio in WT plants and *OE-MsASMT1* transgenic plants. Data are presented as mean ± SE (*n* = 3). The letters indicate the differences between WT and *OE-MsASMT1* transgenic plants according to ANOVA analysis (*p* < 0.05).

Due to our previous study about *HIOMT* gene overexpression in switchgrass regulated flavonoid biosynthesis (Yuan et al., 2016), and to further detect the effects of *MsASMT1* overexpression on flavonoids biosynthesis, we measured the total flavonoids contents in WT plants and *OE-MsASMT1* transgenic plants directly. The results showed that the flavonoid content in all *OE-MsASMT1* transgenic plants was significantly decreased compared to that in WT plants, especially in OE3 and OE25 (Figure 5A). For the stems of *OE-MsASMT1* transgenic alfalfa were thickened, and to explore the effects of melatonin on lignin biosynthesis, we measured the lignin content and lignin composition in WT plants and OE16, OE25 transgenic plants. The lignin content and lignin monomers guaiacyl (G) and syringyl (S) contents showed no significant differences between WT plants and *OE-MsASMT1* transgenic plants (Figures 5B,C), while the S/G ratio was significantly reduced in *OE-MsASMT1* transgenic alfalfa compared to that in WT plants (Figure 5D). The results demonstrated that the increased melatonin content affected flavonoids biosynthesis and lignin monomer composition in phenylalanine metabolism pathway.

### Transcript Profiling Revealed Genes in Phenylalanine Pathway Associated With Flavonoid and Lignin Metabolism Are Changed in *OE-MsASMT1* Transgenic Plants

**FIGURE 6:**
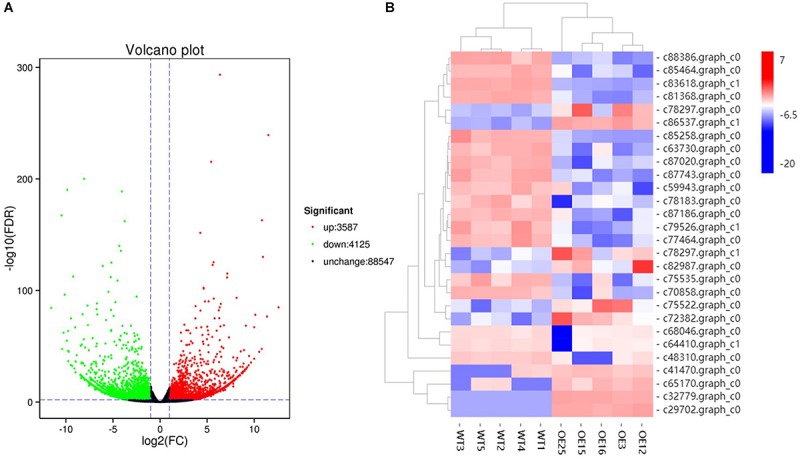
The volcano plot of total differential expression genes (DEGs) **(A)** and heatmap of DEGs associated with flavonoids biosynthesis **(B)** between WT plants and *OE-MsASMT1* transgenic plants.

To gain insight into the underlying molecular mechanism of *MsASMT1* overexpression in plant growth and metabolism, we conducted transcript profiling analysis using RNA-seq with WT plants and *OE-MsASMT1* transgenic plants and five biological replicates were sequenced for each group. A total of 7712 differentially expressed genes (DEGs) were obtained, of which 3587 genes were upregulated, while 4125 genes were downregulated (Figure 6A). GO enrichment analysis indicated that a wide range of DEGs clustered into metabolic process (1329), cellular process (1374), catalytic activity (1306), single-organism process (822), biological regulation (472), response to stimulus (476), signaling (209), transporter activity (130), etc. (Supplementary Figure S5). In the large group of DEGs in metabolic process, a large number of genes in phenylalanine metabolism pathway were altered and 28 of these genes participated in flavonoids biosynthesis and 13 genes participated in lignin biosynthesis and degradation. Some of these genes were identified as UDP-glucosyltransferase family genes, which are involved in flavonoid glycosylation. The heatmap of DEGs related to flavonoid biosynthesis is shown in Figure 6B. We also observed a large amount of genes response to plant hormones, including ethylene (ET), gibberellin (GA), auxin (IAA), abscisic acid (ABA), jasmonic acid (JA) and salicylic acid (SA) biosynthesis and signaling response genes, were altered in *OE-MsASMT1* transgenic alfalfa, which provided evidence of melatonin playing function in plant growth and metabolism by crosstalk with phytohormones. The significant DEGs (|log_2_FC| > 1.5) related to flavonoid and lignin biosynthesis, hormone signaling and defense responses are listed in Supplementary Table S2.

**FIGURE 7:**
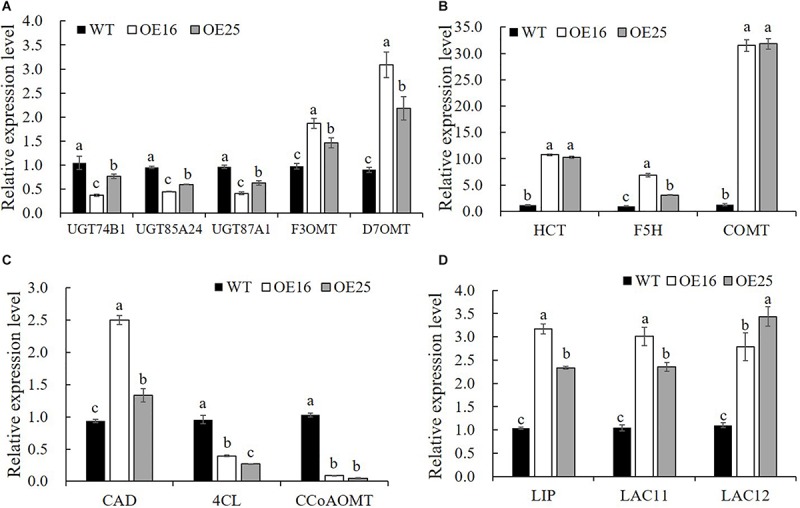
Relative expression levels of genes related to flavonoids biosynthesis **(A)** and lignin biosynthesis **(B–D)** in WT plants and *OE-MsASMT1* transgenic plants. Data are presented as mean ± SE (*n* = 3). The letters indicate the differences between WT and *OE-MsASMT1* transgenic plants according to ANOVA analysis (*p* < 0.05).

To confirm that the overexpression of *MsASMT1* affected the expression of flavonoid biosynthesis genes, we selected 14 DEGs for verification in transgenic plants OE16 and OE25 by qRT-PCR. The results showed that the expression levels of UDP-glucosyltransferase *UGT74B1* (c87186.graph_c0), *UGT85A24* (c83618.graph_c1), and *UGT87A1* (c78183.graph_c0) were significantly decreased in OE16 and OE25, which were involved in the flavonoid glucuronidation process with quercetin 3-O-glucosyltransferase activity and quercetin 7-O-glucosyltransferase activity. Transcripts of *F3OGT* (UDP-glucose flavonoid 3-O-glucosyltransferase; c41470.graph_c0) and *I7OMT* (isoflavone 7-O-methyltransferase; c32779.graph_c0) were obviously upregulated in OE16 and OE25 plants (Figure 7A). Similarly, the transcript levels of several genes involved in lignin biosynthesis were found to be significantly changed in *OE-MsASMT1* transgenic plants. The relative expression levels of *4CL* (4-coumarate: CoA ligase; c67220.graph_c0) and *CCoAOMT* (caffeyl CoA O-methyltransferase; c89170.graph_c0) were downregulated, and the relative expression levels of *CAD* (coniferyl-aldehyde dehydrogenase; c57796.graph_c0), *HCT* (hydroxycinnamoyl transferase; c29702.graph_c0), *COMT* (caffeic acid O-methyltransferase; c71088.graph_c0), and *F5H* (ferulate 5-hydroxylase; c69879.graoh_c0) were significantly upregulated in *OE-MsASMT1* transgenic plants (Figures 7B,C). In addition, the relative expression levels of lignin peroxidase (*LIP*) and laccase (*LAC11*, *LAC12*) genes, which also involved in lignin biosynthesis, were also significantly upregulated in *OE-MsASMT1* transgenic plants compared with WT plants (Figure 7D).

### Metabolic Profiling Revealed Flavonoid Accumulation Changes in *OE-MsASMT1* Transgenic Plants

**FIGURE 8:**
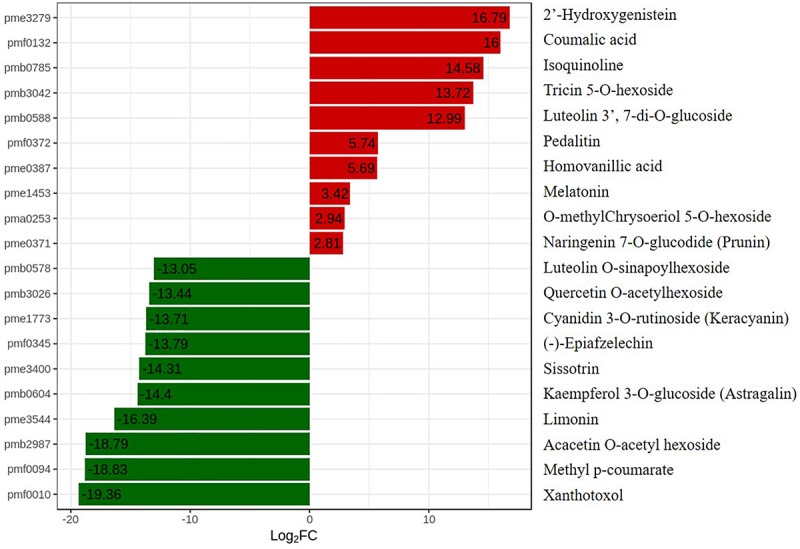
The top ten upregulated and downregulated metabolites between WT plants and *OE-MsASMT1* transgenic plants. Red represent the upregulated metabolites in *OE-MsASMT1* transgenic plants; green represent the downregulated metabolites in *OE-MsASMT1* transgenic plants.

**FIGURE 9:**
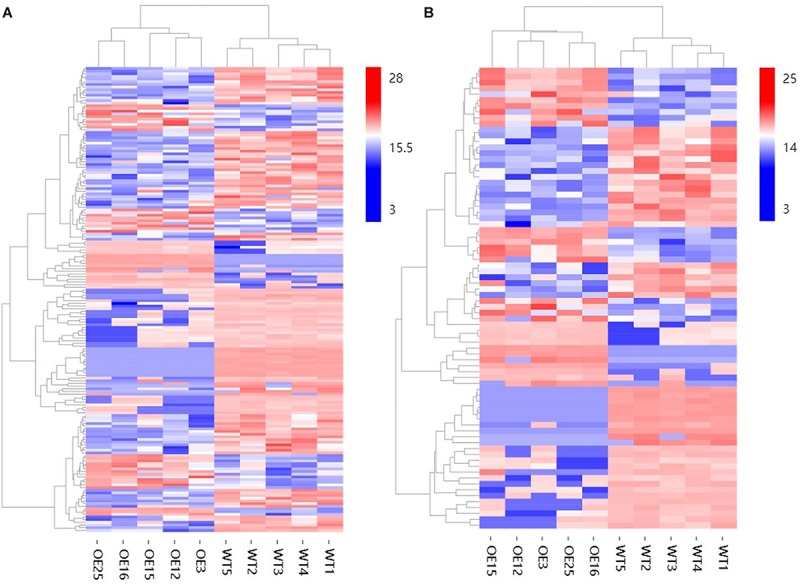
The heatmaps of total differential metabolites **(A)** and the differential metabolites belong to flavonoids **(B)** between WT plants and *OE-MsASMT1* transgenic plants.

To further explore changes in compounds that accompanied changes in gene regulation, a widely targeted metabolomics assay was performed with WT plants and *OE-MsASMT1* transgenic plants. A total of 174 differential secondary metabolites were identified, in which 120 metabolites were decreased and 54 were increased in *OE-MsASMT1* transgenic plants. The top 10 upregulated and downregulated metabolites are shown in Figure 8. There were 78 flavonoid metabolites among these 174 identified metabolites, including 37 flavones, 12 flavonols, 6 flavanones, 6 isoflavones, 8 anthocyanins, and 9 flavonoids. The heatmaps of the total differential metabolites (Figure 9A) and the flavonoid metabolites (Figure 9B) compared between WT plants and *OE-MsASMT1* transgenic plants are shown in Figure 9. The concentrations of these significantly decreased and increased flavonoid metabolites (|log_2_FC| > 1.5) in *OE-MsASMT1* transgenic plants are listed in Supplementary Tables S3, S4, respectively. Quercetin 7-O-malonylhexosyl-hexoside (-12.9-fold), quercetin O-acetylhexoside (-13.4-fold), quercetin 4’-O-glucoside (-4.01-fold) and quercetin 3-alpha-L-arabinofuranoside (-3.53-fold), all of which were quercetin glycoside derivative, were significantly decreased in *OE-MsASMT1* transgenic plants. The concentration of kaempferol 3-O-glucoside, belonging to kaempferol glycoside derivates, in *OE-MsASMT1* transgenic plants was 14.4-fold lower than that in WT plants. Furthermore, the concentrations of formononetin (-3.1-fold), formononetin 7-O-glucoside (-4.23-fold) and biochanin 7-O-glucoside (-14.3-fold) were also decreased in *OE-MsASMT1* transgenic plants (Supplementary Table S3). However, the concentrations of chrysoeriol derivates including chrysoeriol O-hexosyl-O-hexoside (1.52-fold), chrysoeriol O-glucuronic acid-O-hexoside (1.51-fold), O-methylChrysoeriol 5-O-hexoside (2.94-fold) and O-methylChrysoeriol 7-O-hexoside (2.42-fold) were increased in *OE-MsASMT1* transgenic plants (Supplementary Table S4), and the concentrations of naringenin derivates naringenin C-hexoside (1.88-fold) and naringenin 7-O-glucoside (2.81-fold), tricin (2.06-fold) and its derivates tricin 5-O-hexoside (13.7-fold), tricin 7-O-hexoside (1.98-fold), and tricin 5-O-feruloylhexoside (1.73-fold) were increased in *OE-MsASMT1* transgenic plants (Supplementary Table S4). In addition, there were also some other metabolites belong to alkaloids, terpene, polyphenol and phenylpropanoids were changed in *OE-MsASMT1* transgenic plants, which might be responsible to plants response to environmental stimulus. Limonin, a compound of highly oxidized triterpenoids and a source of bitterness, potentially functions in preventing diseases and decreased almost 16.4-fold in *OE-MsASMT1* plants. The concentrations of xanthotoxol (a natural furanocoumarins with various biological activities) and methyl *p*-coumarate (an esterified derivative of *p*-coumaric acid) in *OE-MsASMT1* transgenic plants were -19.4 and -18.8 folds, respectively, to WT plants (Supplementary Table S5).

### Overexpression of *MsASMT1* Affects Forage Quality

To evaluate the effects of *MsASMT1* on forage quality, the shoots of WT plants and *OE-MsASMT1* transgenic plants at the early flowering stage were harvested for forage quality evaluation. The overexpression of *MsASMT1* led to a significant increase in acid detergent fiber content (ADF) and neutral detergent fiber content (NDF). The increases of NDF and ADF contents in *OE-MsASMT1* transgenic plants varied from 1.8% to 4.0% and from 1.0% to 3.9%, respectively, compared to those in WT plants. The cellulose contents in *OE-MsASMT1* transgenic plants were also significantly increased (Table 3). Other nutritive quality traits, including ADL and hemicellulose, did not show obvious changes between *OE-MsASMT1* transgenic plants and WT plants.

**TABLE 3:** The forage quality of WT and *OE-MsASMT1* transgenic alfalfa.

**Lines**	**NDF/%**	**ADF/%**	**ADL/%**	**Hemicellulose/%**	**Cellulose/%**
WT	47.73 ± 0.45b	31.29 ± 0.55c	10.96 ± 0.69	16.43 ± 0.28	20.34 ± 0.25c
OE3	49.78 ± 1.03ab	32.28 ± 0.18bc	9.63 ± 0.43	17.50 ± 1.19	22.65 ± 0.27b
OE12	50.01 ± 0.79ab	33.89 ± 0.98ab	10.09 ± 0.53	16.89 ± 0.56	22.89 ± 0.33ab
OE15	49.56 ± 0.60ab	34.17 ± 1.22ab	11.31 ± 1.25	15.39 ± 0.69	22.86 ± 0.03ab
OE16	51.78 ± 0.94a	34.88 ± 0.88a	10.97 ± 1.41	16.90 ± 1.70	23.91 ± 0.59a
OE25	50.94 ± 1.02ab	35.16 ± 0.76a	11.97 ± 0.94	15.79 ± 1.49	23.18 ± 0.20ab

*Each value represents the mean ± SE from three replicates. The letter following the value in the same row indicates the differences between WT plants and *OE-MsASMT1* transgenic plants according to ANOVA analysis (*p* < 0.05).*

## Discussion

Melatonin, a bioactive molecule with multiple functions, is ubiquitously present in various plant species. For the reported melatonin biosynthesis pathway, six genes were implicated in the biosynthesis of melatonin in plants (Back et al., 2016). *SNAT* and *ASMT* are associated with the conversion of serotonin to melatonin, which are the rate-limiting steps for melatonin biosynthesis. In our previous study, we ectopically expressed *AANAT* and *HIOMT* from *Ovis aries* in switchgrass, which led to an increase in the melatonin content, promoted plant growth and development, and improved salt tolerance in transgenic plants (Huang et al., 2017). The overexpression of *HIOMT* regulated the phenylalanine metabolism pathway in transgenic switchgrass, especially flavonoid biosynthesis (Yuan et al., 2016), while the regulation effects and mechanisms have not been studied. The function of melatonin and the genes participating in melatonin biosynthesis were conserved, but there might be some fine distinction among different plant species. Alfalfa is an important forage grass with a rich nutritional value and powerful health-promoting properties. Taking advantage of the health-promoting properties of melatonin, the forage quality improvement of alfalfa has profound implications.

To obtain alfalfa with a high melatonin level, we successfully isolated an *ASMT* gene from the alfalfa genome according to the reported gene sequences in other plant species. The melatonin contents in *OE-MsASMT1* transformants were significantly increased and were positively correlated with the transcript levels of *MsASMT1*. The expression of other genes related to melatonin biosynthesis, including *MsTDC, MsT5H, MsSNAT*, and *MsCOMT*, was upregulated in *MsASMT1* overexpression lines, leading to increased melatonin contents in *OE-MsASMT1* transgenic plants. In our study, the circadian rhythm analysis results showed that the peaks of melatonin levels and *MsASMT1* expression levels in alfalfa throughout one day weren’t at the same time. *MsASMT1* expression and the melatonin content all have two peaks during one day, while there were no regular relationships between *MsASMT1* expression level and melatonin content, which might result from the endogenous melatonin content is regulated by all genes in the melatonin biosynthesis pathway, and the biosynthesis of metabolites is always later than the transcripts of genes.

The exogenous or endogenous regulation of melatonin promoted plant growth and development (Byeon and Back, 2014), which also occurred in alfalfa. *OE-MsASMT1* transgenic alfalfa exhibited a consistent phenotype, they were more vigorous growth, possessed larger leaves, thicker stems and grew faster than WT plants, all of which was accompanied by bushy branches at the early vegetative stage. The vigorous stems of *OE-MsASMT1* transgenic plants might arise from the highly specific expression pattern of *MsASMT1* in alfalfa. Presumably related to the quantification of auxin, *OE-MsASMT1* stems grew faster, and the internode length also increased rapidly. Due to the consistent phenotype, we only chose OE16 and OE25 with low and moderate expression level of *MsASMT1* gene respectively to measure the IAA content and the relative expression level of genes related to branch formation to reflect the difference between *OE-MsASMT1* transgenic plants and WT plants. Compared to cells of stems in WT plants, cells of stems in *OE-MsASMT1* transgenic alfalfa were irregularly arranged, and the intercellular spaces were larger, with several hollow areas existing in the cell population, which might result from a strong mechanical strength between cells of stems for rapid growth of thick stems.

Flavonoids are important secondary metabolites in plants, which have versatile physiological functions in plant growth and development. Previous studies reported that flavonoids inhibited the biosynthesis of melatonin (Lee et al., 2018). In our study, melatonin-rich transgenic alfalfa produced less flavonoids compared to WT plants, which suggested that melatonin in turn inhibited the biosynthesis of flavonoids, especially quercetin, kaempferol, formononetin, and biochanin. For flavonoids playing an important role in defending against pathogen/insect attacks, the reduced flavonoid content in *OE-MsASMT1* transgenic alfalfa might lead transgenic plants sensitive to pathogen/insect attacks. However, many studies have shown that the exogenous application or endogenous improvement of melatonin enhances various abiotic or biotic stress tolerances (Shi et al., 2015; Antoniou et al., 2017; Mandal et al., 2018; Liu et al., 2019). To test whether the elevated melatonin or the depressed flavonoids had a greater effect on stress resistance of *OE-MsASMT1* transgenic alfalfa, we will select several abiotic or biotic stresses to detect the adverse defense ability of *OE-MsASMT1* transgenic alfalfa in the future. Furthermore, in our RNA-seq data, we found a large cluster of genes related to plants defense responses and disease resistance were changed in *OE-MsASMT1* transgenic alfalfa, which also portended that the defense responses might alter in *OE-MsASMT1* transgenic plants.

**FIGURE 10:**
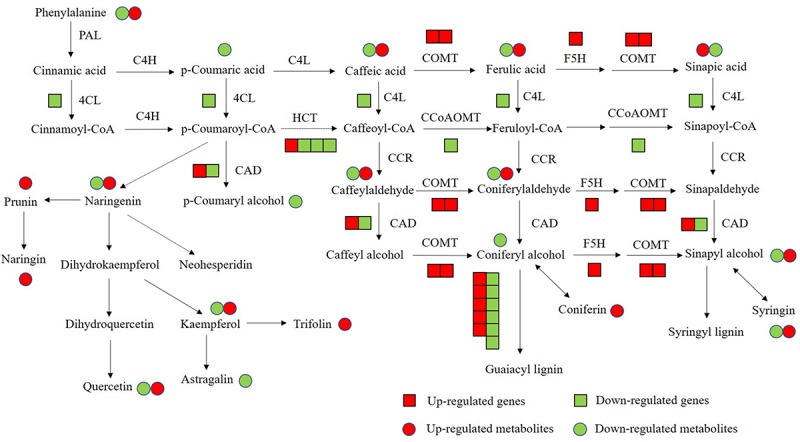
The conjoint analysis of genes and metabolites changed in phenylalanine pathway between WT plants and *OE-MsASMT1* transgenic plants. The square represents changed genes, the circle represents changed metabolites. And red color represents the up-regulated genes or metabolites, green represents the down-regulated genes or metabolites.

Flavonoids and lignin are major metabolites in phenylalanine pathway. According to our transcriptomic and metabolomic data, a large number of genes clustered into flavonoids and lignin biosynthesis and many metabolites belong to the precursors of flavonoids and lignin were changed (Figure 10). Among these genes in flavonoids biosynthesis, a large subgroup of genes are UDP-glucosyltransferase family genes, which is involved in flavonoid glycosylation, the major modification in flavonoid biosynthesis (Jones and Vogt, 2001; Yin et al., 2017). And many genes clustered into the MYB transcription factor family. R2R3-MYB transcription factors are widely involved in the phenylalanine metabolic pathway and play an important role in regulating flavonoid biosynthesis and stress responses in plants (Liu et al., 2015; Yan et al., 2015). There are 36 MYB transcription factors were changed in *OE-MsASMT1* transgenic alfalfa, and 16 of them were downregulated, such as MYB4 and MYB14. The significantly changed (| log_2_FC| > 1.5) MYB transcription factors between *OE-MsASMT1* transgenic plants and WT plants were listed in Supplementary Table S2. The significant downregulation of these genes might also be involved in the decreased flavonoids content in *OE-MsASMT1* transgenic alfalfa. Lignin is a major component of plant secondary cell wall and is essential for mechanical support, water transport and defense responses (Boerjan et al., 2003; Rao et al., 2019). In our report, the transcripts of many genes involved in lignin biosynthesis were altered. And the contents of two lignin alcohol monomers coniferyl alcohol and *p*-coumaryl alcohol in the metabolomics results were significantly decreased in *OE-MsASMT1* transgenic plants. Then, due to the complexity of lignin measurement and the same principle with IAA detection, we only chose OE16 and OE25 plants to quantify the lignin content and lignin monomer composition, and found that the lignin content and lignin monomers (G and S) contents showed no significant differences between WT plants and OE16, OE25 transgenic plants, while the S/G ratio was decreased in *OE-MsASMT1* transgenic plants which might influence the saccharification efficiency in transgenic plants. The results indicated that melatonin plays a role in regulating flavonoids and lignin biosynthesis and metabolism. To further explain the function of *MsASMT1* in alfalfa, transgenic plants with suppressed expression of the endogenous *ASMT* gene through RNAi or CRISPR-Cas9 would be generated to further explore the function of melatonin in alfalfa growth and secondary metabolism.

## Conclusion

In summary, the overexpression of *MsASMT1* improved endogenous melatonin levels in transgenic alfalfa. *OE-MsASMT1* transgenic alfalfa grew faster, had larger leaves and robust stems with increased cell sizes, cell numbers and vascular bundles. Nevertheless, *OE-MsASMT1* transgenic alfalfa with rich melatonin accumulated less flavonoids, mainly quercetin, kaempferol, formononetin, and biochanin on account of the downregulation of genes associated with flavonoid biosynthesis. In this report, we found that melatonin plays a role in regulating flavonoids and lignin biosynthesis and inhibits flavonoids biosynthesis in alfalfa. We first reported the effects of endogenous melatonin on alfalfa plant growth and metabolism. This report provides insight into the function of melatonin in plant secondary metabolism and broadens the biological function of endogenous melatonin in plants.

## Data Availability Statement

The datasets generated for this study can be found in the GenBank accession number: MN092350, NCBI Sequence Read Archive (SRA) database accession number: PRJNA555673.

## Author Contributions

HC and YZ contributed the conception and design of the study. HC, TW, and HL performed the experiments. DT, XL, and XC organized the database. CG, HZ, and ML performed the statistical analysis. HC wrote the first draft of the manuscript. HW and YZ revised the manuscript. All authors read and approved the submitted manuscript.

## Conflict of Interest

The authors declare that the research was conducted in the absence of any commercial or financial relationships that could be construed as a potential conflict of interest.

## Footnotes

1 https://www.ncbi.nlm.nih.gov/Structure/cdd/wrpsb.cgi

2 https://web.expasy.org/protparam/

3 https://submit.ncbi.nlm.nih.gov/subs/sra/
