# Supplementary material for: Overexpression of MsASMT1 Promotes Plant Growth and Decreases Flavonoids Biosynthesis in Transgenic Alfalfa (Medicago sativa L.)
Source: Front Plant Sci. 2020 Apr 28;11:489. doi: 10.3389/fpls.2020.00489 (PMC7199503; doi:10.3389/fpls.2020.00489)
Supplement: Supplementary file 1 [file Data_Sheet_1.docx]

Table S1 Primers lists

| **Primer name** | **Primer sequence (5’-3’)** | **Description** |
| --- | --- | --- |
| MtASMT-F | GCAAGTAAGTATGGAATCCCAAAAT | Gene clone |
| MtASMT-R | TTATGGATAGATCTCAATCATGGAC |  |
| ASMT-F | CGCGGATCCGCAAGTAAGTATGGAATCCCAAAAT | Add enzyme cutting site |
| ASMT-R | CGGGGTACCTTATGGATAGATCTCAATCATGGA |  |
| MsASMT1-F | ATTTCTTCACTACCAATCCACCC | RT-PCR、qRT-PCR for ASMT |
| MsASMT1-R | CCACACTCATTGGATTGTTCTAAA |  |
| Actin-F | CAAAAGATGGCAGATGCTGAGGAT | Reference gene |
| Actin-R | CATGACACCAGTATGACGAGGTCG |  |
| SL-F | CAGTGGTCTCACAACATGGAATCCCAAAATGGAGA | Vector construction for subcellular localization |
| SL-R | CAGTGGTCTCATACATGGATAGATCTCAATCATGG |  |
| TDC-F | CTCGCAGGATCTTGTCACGG | qRT-PCR for TDC |
| TDC-R | AGGCACTCCTTCTGCCTCAT |  |
| T5H-F | CATTGTTCTTCTGCGTCGCC | qRT-PCR for T5H |
| T5H-R | CCAAGTGCCACCCTGCATAC |  |
| SNAT-F | GTCAGAGGGGAATGAACAAAA | qRT-PCR for SNAT |
| SNAT-R | TTCCACGACTTTACTATCTGCG |  |
| COMT-F | GGCCACCACCAACATCAACA | qRT-PCR for COMT |
| COMT-R | CCCAGCTTAGACTTGAGGTTCA |  |
| UGT78A1-F | TACCAACATCAACAGGCCACTGC | qRT-PCR for UGT78A1 |
| UGT78A1-R | CCAACGTGGTTCCACCAGAGC |  |
| UGT74B1-F | AGAAGAGCTGGCTTTGGGAT | qRT-PCR for UGT74B1 |
| UGT74B1-R | TCCAACAGCATCATGTGCCA |  |
| UGT85A24-F | GCCTGGCACCCAATCTATGG | qRT-PCR for UGT85A24 |
| UGT85A24-R | TGGCTTCATGGCCTACATGC |  |
| F3OGT-F | GAGCGGACAGCAGAGATTGGAAG | qRT-PCR for F3OGT |
| F3OGT-R | TGAATTCCAACCACAATGCGAAGC |  |
| I7OMT-F | TCTGTTTCAAGCAAGGGCAGA | qRT-PCR for I7OMT |
| I7OMT-R | ACCCTGCTTCAACGAAGAGC |  |
| 4CL-F | TACGCGCAGCTAAGGAGAGGAAG | qRT-PCR for 4CL |
| 4CL-R | AGCAAGAATCATCGGTGGCACAAC |  |
| CAD-F | TTCGTCGGCGACCATTGTGTC | qRT-PCR for CAD |
| CAD-R | TGGTTCAATCCTGCCACTCATGC |  |
| HCT-F | ACTGTTACGGCATTTTGGCGA | qRT-PCR for HCT |
| HCT-R | GCTCGGATTGCATGTGGTGA |  |
| F5H-F | GGTCAGGAAGAAGGTCATGTCCAG | qRT-PCR for F5H |
| F5H-R | AGCAACAAGCTGAACAGCTCTAGG |  |
| CCoAOMT-F | GCTCAACCACCCTCTTGTGA | qRT-PCR for CCoAOMT |
| CCoAOMT-R | ACAAGGACCTGCTCTCCCTC |  |
| LIP-F | CCGGTGGTCCGTTCTATCCT | qRT-PCR for LIP |
| LIP-R | AGGGTGAAGGAAGCTCATCTGT |  |
| LAC11-F | GCGTGCCACCATAGTGTTCCTC | qRT-PCR for LAC11 |
| LAC11-R | GCGGACGGACCAGCTTATATCAC |  |
| LAC12-F | CCTACCGGCAACTTGGTTGG | qRT-PCR for LAC12 |
| LAC12-R | GCGGGTCACAACATGACAGT |  |

Table S2 Selected DEGs related to flavonoid and lignin biosynthesis, MYB transcription factors, hormone signaling and defense responses

| **Gene ID** | **Gene name** | **Gene description** | **Log_2_FC** | **Regulated** |
| --- | --- | --- | --- | --- |
| c29702.graph_c0 | SHT | Spermidine hydroxycinnamoyl transferase | 4.82 | up |
| c32779.graph_c0 | D7OMT | Isoflavone 7-O-methyltransferase | 3.86 | up |
| c65170.graph_c0 | UGT78G1 | Flavonoid 3-O-glucosyltransferase | 3.40 | up |
| c41470.graph_c0 | GT2 | Anthocyanidin 3-O-glucosyltransferase 2 | 2.66 | up |
| c75522.graph_c0 | UGT83A1 | UDP-glycosyltransferase 83A1 | 2.24 | up |
| c86537.graph_c1 | UGT73C1 | UDP-glycosyltransferse 73C1 | 2.26 | up |
| c78297.graph_c1 | CYP75B1 | Flavonoid 3-monooxygenase | 2.18 | up |
| c72382.graph_c0 | UGT72Z1 | Hydroquinone glucosyltransferase | 1.82 | up |
| c82987.graph_c0 | CYP736A12 | Cytochrome P450 | 1.81 | up |
| c88948.graph_c0 | CCR1 | Cinnamoyl-CoA reductase 1 | -6.38 | down |
| c83618.graph_c1 | UGT85A24 | UDP-glucosyltransferase 85A24 | -3.74 | down |
| c48310.graph_c0 | HST | Shikimate O-hydroxycinnamoyl transferase | -2.85 | down |
| c70858.graph_c0 | VR | Vestitone reductase | -2.34 | down |
| c77464.graph_c0 | SHT | Spermidine hydroxycinnamoyl transferase | -2.18 | down |
| c87186.graph_c0 | UGT74B1 | UDP-glycosyltransferase 74B1 | -2.04 | down |
| c85464.graph_c0 | UGT72B1 | UDP-glycosyltransferase 72B1 | -1.78 | down |
| c68046.graph_c0 | UGT85A3 | UDP-glycosyltransferase 85A3 | -1.75 | down |
| c87020.graph_c0 | UGT89B1 | UDP-glycosyltransferase 89B1 | -1.72 | down |
| c64410.graph_c1 | UGT85K4 | Linamarin synthase 1 | -1.69 | down |
| c88386.graph_c0 |  | UDP-glucosyltransferase family protein | -1.67 | down |
| c63730.graph_c0 | CCoAOMT | Caffeoyl-CoA 3-O-methyltransferase | -1.53 | down |
| c59943.graph_c0 |  | UDP-glucosyltransferase family protein | -1.51 | down |
| c80659.graph_c0 | LAC2 | Laccase-2 | 1.68 | up |
| c87071.graph_c0 | LAC11 | Laccase-11 | 3.66 | up |
| c87071.graph_c1 | LAC11 | Laccase-11 | 3.84 | up |
| c69879.graph_c0 | CYP84A1 | Cytochrome P450 84A1 | 2.36 | up |
| c56980.graph_c0 | COMT | Caffeic acid 3-O-methyltransferase | 3.11 | up |
| c71088.graph_c0 | COMT | Caffeic acid 3-O-methyltransferase | 3.37 | up |
| c67220.graph_c0 | PER48 | Putative Peroxidase 48 | 2.30 | up |
| c57796.graph_c0 | ALDH2B4 | Aldehyde dehydrogenase family 2 member B4 | -6.34 | down |
| c73245.graph_c0 | CAD | Cinnamyl-alcohol dehydrogenase | -4.20 | down |
| c68772.graph_c0 | CAD-like | Cinnamyl alcohol dehydrogenase-like protein | 2.69 | up |
| c79001.graph_c4 | MYB4 | MYB transcription factor | -6.74 | down |
| c47110.graph_c0 |  | MYB/SANT-like DNA-binding domain protein | -5.78 | down |
| c32763.graph_c0 |  | MYB-like DNA-binding domain protein | -5.01 | down |
| c37438.graph_c1 |  | MYB/SANT-like DNA-binding domain protein | -4.90 | down |
| c95195.graph_c0 | GT-3b | MYB/SANT-like DNA-binding domain protein | -4.58 | down |
| c92287.graph_c0 |  | MYB-like DNA-binding domain protein | -4.34 | down |
| c60298.graph_c0 |  | MYB-like DNA-binding domain protein | -3.92 | down |
| c93306.graph_c0 |  | MYB-like DNA-binding domain protein | -3.83 | down |
| c70265.graph_c0 |  | MYB-like DNA-binding domain protein | -3.12 | down |
| c84787.graph_c0 |  | MYB/SANT-like DNA-binding domain protein | -2.01 | down |
| c80667.graph_c0 | MYB30 | MYB transcription factor | -1.70 | down |
| c43181.graph_c0 |  | MYB/SANT-like DNA-binding domain protein | -1.65 | down |
| c50751.graph_c0 |  | MYB-like DNA-binding domain protein | 8.20 | up |
| c73219.graph_c0 |  | MYB/SANT-like DNA-binding domain protein | 7.35 | up |
| c64539.graph_c0 | LIMYB | L10-interacting MYB domain-containing protein | 6.80 | up |
| c76830.graph_c0 |  | MYB/SANT-like DNA-binding domain protein | 5.46 | up |
| c60393.graph_c0 |  | MYB-like DNA-binding domain protein | 5.04 | up |
| c60786.graph_c0 |  | MYB/SANT-like DNA-binding domain protein | 4.77 | up |
| c59661.graph_c0 |  | MYB transcription factor | 4.23 | up |
| c37203.graph_c0 | GT-4 | MYB/SANT-like DNA-binding domain protein | 3.69 | up |
| c78042.graph_c0 | TT2 | Tannin-related R2R3 MYB transcription factor | 3.62 | up |
| c90260.graph_c0 |  | MYB-like DNA-binding domain protein | 2.40 | up |
| c63112.graph_c0 | MYB3R-3 | Transcription factor MYB3R-3 | 2.03 | up |
| c56630.graph_c0 | EIL3 | Ethylene insensitive 3-like 3 protein | 4.35 | up |
| c90832.graph_c0 | EIL5 | Ethylene insensitive 3-like 5 protein | 4.13 | up |
| c89647.graph_c0 | EIN3 | Ethylene insensitive 3 protein | 3.94 | up |
| c82125.graph_c4 | WIN1 | Ethylene-responsive transcription factor | 2.22 | up |
| c84866.graph_c2 | RAP2-7 | Ethylene-responsive transcription factor | 2.06 | up |
| c73283.graph_c0 | ERF1B | Ethylene-responsive transcription factor 1B | -1.67 | down |
| c42680.graph_c0 |  | Auxin-induced 5NG4-like protein | 5.45 | up |
| c32815.graph_c0 | BIG | Auxin transport protein BIG | 3.18 | up |
| c59400.graph_c0 | ARF6 | Auxin response factor 6 | 3.03 | up |
| c78951.graph_c3 |  | Auxin-responsive family protein, putative | -6.82 | down |
| c90535.graph_c0 | ABP19B | Auxin-binding protein | -3.57 | down |
| c86480.graph_c1 | ZEP | Zeaxanthin epoxidase | 1.62 | up |
| c86892.graph_c0 |  | 9-cis-epoxycarotenoid dioxygenase | 1.54 | up |
| c88288.graph_c1 | GA20ox1 | Gibberellin 20 oxidase 1-like protein | 5.74 | up |
| c79028.graph_c0 | GA20ox2 | Gibberellin 20 oxidase 2 | 2.45 | up |
| c66846.graph_c0 | GID1 | Gibberellin receptor GID1, putative | 1.85 | up |
| c68816.graph_c1 | GA2ox8 | Gibberellin 2-beta-dioxygenase 8 | -4.54 | down |
| c81773.graph_c0 | GID1 | Gibberellin receptor GID1, putative | -2.00 | down |
| c78029.graph_c0 | GA2ox8 | Gibberellin 2-beta-dioxygenase 8 | -1.58 | down |
| c75733.graph_c1 | COI1 | Coronatine-insensitive protein 1 | 7.43 | up |
| c79446.graph_c0 |  | Jasmonate O-methyltransferase | 2.89 | up |
| c66280.graph_c0 | COI1 | Coronatine-insensitive protein 1 | -5.72 | down |
| c83278.graph_c3 |  | Regulation of salicylic acid signaling pathway | 4.52 | up |
| c79289.graph_c2 |  | Response to salicylic acid | -2.83 | down |
| c65715.graph_c0 | PAZ1C | Serpin-Z1C | 1.98 | up |
| c46327.graph_c1 | PAZXA | Serpin-ZXA | -4.69 | down |
| c29214.graph_c0 | PAZ2A | Serpin-Z2A | -4.90 | down |
| c46327.graph_c0 | PAZX | Serpin-ZX | -5.99 | down |
| c92365.graph_c0 | PAZ10 | Serpin-Z10 | -3.52 | down |
| c31167.graph_c0 | PAZ7 | Serpin-Z7 | -5.79 | down |
| c66974.graph_c0 | PAZX | Serpin-ZX | -5.53 | down |
| c88948.graph_c0 | CCR1 | Cinnamoyl-CoA reductase 1 | -6.38 | down |
| c67405.graph_c0 | CXE18 | Probable carboxylesterase 18 | 4.52 | up |
| c66846.graph_c0 | CXE15 | Probable carboxylesterase 15 | 1.85 | up |
| c74495.graph_c0 | CXE17 | Probable carboxylesterase 17 | 1.52 | up |
| c30369.graph_c0 | HVA22J | HVA22-like protein | 4.12 | up |
| c50705.graph_c0 | MIRO1 | Mitochondrial Rho GTPase 1 | 4.84 | up |
| c88918.graph_c0 | MIRO2 | Mitochondrial Rho GTPase 2 | 4.10 | up |
| c49447.graph_c0 |  | B-cell receptor-associated protein 31-like | 2.76 | up |
| c89003.graph_c0 | MIF | Macrophage migration inhibitory factor | 2.69 | up |
| c88895.graph_c0 | MIF | Macrophage migration inhibitory factor | 2.12 | up |
| c31458.graph_c0 |  | Mitochondrial glycoprotein | 1.93 | up |
| c79013.graph_c1 | GCR2 | G protein coupled receptor | -1.68 | down |
| c81773.graph_c0 | CXE1 | Carboxylesterase 1 | -1.99 | down |
| c50171.graph_c0 | CXE18 | Probable carboxylesterase 18 | -2.18 | down |
| c95365.graph_c0 |  | B-cell receptor-associated protein 31-like | -3.56 | down |
| cc77174.graph_c0 |  | Macrophage migration inhibitory factor | -4.00 | down |
| c93211.graph_c0 |  | LMBR1-like membrane protein | -4.38 | down |
| c89960.graph_c0 |  | Mitochondrial glycoprotein | -4.70 | down |
| c89389.graph_c0 |  | Inhibitor of apoptosis-promoting Bax1 | -5.59 | down |

Note: The regulated types of up and down represent the genes were upregulated or downregulated respectively in *OE-MsASMT1* plants compared to that in WT plants. The DEGs listed in the table were |log_2_FC|>1.5.

Table S3 Down-regulated metabolites belong to flavonoids in *OE-MsASMT1* transgenic plants compared to WT plants

| **Index** | **Compounds** | **Class** | **Log_2_FC** |
| --- | --- | --- | --- |
| pmb0709 | Quercetin 7-O-malonylhexosyl-hexoside | Flavonol | -12.9 |
| pmb3026 | Quercetin O-acetylhexoside | Flavonol | -13.4 |
| pme3129 | Quercetin 4’-O-glucoside (Spiraeoside) | Flavonol | -4.01 |
| pme0361 | Quercetin 3-alpha-L-arabinofuranoside (Avicularin) | Flavonol | -3.53 |
| pmb0604 | Kaempferol 3-O-glucoside (Astragalin) | Flavonol | -14.4 |
| pmb0565 | Syringetin 3-O-hexoside | Flavonol | -3.56 |
| pme1502 | Kumatakenin | Flavonol | -2.44 |
| pma0214 | Methyl-quercetin O-hexoside | Flavonol | -2.25 |
| pme1498 | Formononetin (4'-O-methyldaidzein) | Isoflavone | -3.10 |
| pme3504 | Formononetin 7-O-glucoside (Ononin) | Isoflavone | -4.23 |
| pme3400 | Biochanin7-O-glucoside (Sissotrin) | Isoflavone | -14.3 |
| pme1587 | Daidzein 7-O-glucoside (Daidzin) | Isoflavone | -1.67 |
| pme0204 | Amentoflavone | Flavone | -11.8 |
| Pmb2984 | Acetyl-eriodictyol O-hexoside | Flavone | -12.6 |
| pmb2987 | Acacetin O-acetyl hexoside | Flavone | -18.8 |
| pmb0578 | Luteolin O-sinapoylhexoside | Flavone | -13.0 |
| pma6499 | Limocitrin O-hexoside | Flavone | -3.39 |
| pmb0684 | C-hexosyl-luteolin O-sinapic acid | Flavone | -3.08 |
| pmb2978 | Apigenin O-hexosyl-O-pentoside | Flavone | -2.96 |
| pmb1108 | Luteolin 6-C-hexoside 8-C-hexosyl-O-hexoside | Flavone | -2.85 |
| pmb0620 | Chrysoeriol 6-C-hexoside 8-C-hexoside-O-hexoside | Flavone | -2.61 |
| pmb0569 | Syringetin 5-O-hexoside | Flavone | -2.24 |
| pmb0615 | Hesperetin-C-hexosyl-O-hexosyl-O-hexoside | Flavone | -2.19 |
| pmb0639 | 8-C-hexosyl-apigenin O-hexosyl-O-hexoside | Flavone | -2.17 |
| pmb0602 | Syringetin 7-O-hexoside | Flavone | -2.11 |
| Pmb0629 | Chrysoeriol 6-C-hexoside | Flavone | -1.57 |
| pmb3044 | Tricin di-O-hexoside | Flavone | -1.51 |
| pme1773 | Cyanidin 3-O-rutinoside (Keracyanin) | Anthocyanins | -13.70 |
| pme1786 | Malvidin 3,5-diglucoside (Malvin) | Anthocyanins | -2.20 |
| pme0443 | Malvidin 3-O-galactoside | Anthocyanins | -1.72 |
| pme0444 | Malvidin 3-O-glucoside (Oenin) | Anthocyanins | -1.67 |
| pmf0471 | Apiin | Flavonoid | -2.94 |
| pmf0179 | Narcissoside | Flavonoid | -2.28 |
| Pme3217 | Isoliquiritigenin | Flavanone | -1.84 |

Note: The metabolites listed in the table were fold change |Log_2_FC|>1.5.

Table S4 Up-regulated metabolites belong to flavonoids in *OE-MsASMT1* transgenic plants compared to WT plants

| **Index** | **Compounds** | **Class** | **Log_2_FC** |
| --- | --- | --- | --- |
| pme3279 | 2'-Hydroxygenistein | Isoflavone | 16.8 |
| pme1568 | Orobol (5,7,3',4'-tetrahydroxyisoflavone) | Isoflavone | 2.62 |
| pmb3042 | Tricin 5-O-hexoside | Flavone | 13.7 |
| pmb0588 | Luteolin 3',7-di-O-glucoside | Flavone | 13.0 |
| pma0249 | Selgin 5-O-hexoside | Flavone | 2.24 |
| pma0253 | O-methylChrysoeriol 5-O-hexoside | Flavone | 2.94 |
| pme0324 | Chrysin | Flavone | 2.30 |
| pma6638 | O-methylChrysoeriol 7-O-hexoside | Flavone | 2.42 |
| pmb2850 | Tricin | Flavone | 2.06 |
| pma0724 | Naringenin C-hexoside | Flavone | 1.88 |
| pmb0736 | Tricin 7-O-hexoside | Flavone | 1.98 |
| pmb0732 | Tricin 5-O-feruloylhexoside | Flavone | 1.73 |
| pmf0011 | Apigenin 6,8-C-diglucoside | Flavone | 1.55 |
| pmb0603 | Chrysoeriol O-hexosyl-O-hexoside | Flavone | 1.52 |
| pmb0587 | Chrysoeriol O-glucuronic acid-O-hexoside | Flavone | 1.51 |
| pmf0382 | 5,7-Dihydroxy-3',4',5'-trimethoxyflavone | Flavonoid | 2.73 |
| pmf0372 | Pedalitin | Flavonoid | 5.74 |
| pmf0584 | Phloridzin | Flavonoid | 1.93 |
| pme0371 | Naringenin 7-O-glucoside (Prunin) | Flavanone | 2.81 |
| pma1590 | Peonidin O-hexoside | Anthocyanins | 1.78 |

Note: The metabolites listed in the table were fold change (Log_2_FC)>1.5.

Table S5 Significantly changed metabolites except flavonoids in *OE-MsASMT1* transgenic plants compared to WT plants

| **Index** | **Components** | **Class** | **Log_2_FC** | **Type** |
| --- | --- | --- | --- | --- |
| pmb0785 | Isoquinoline | Alkaloids | 14.6 | up |
| pme1453 | Melatonin | Alkaloids | 3.42 | up |
| pmf0461 | Febrifugine | Alkaloids | 2.07 | up |
| pma6270 | sn-Glycero-3-phosphocholine | Alkaloids | -2.17 | down |
| pme2155 | Theobromine | Alkaloids | -2.12 | down |
| pmf0559 | Abrine | Alkaloids | -2.21 | down |
| pme3544 | Limonin | Terpene | -16.4 | down |
| pme0080 | Cucurbitacin B | Terpene | -2.90 | down |
| pmf0315 | Sclareol | Terpene | -2.25 | down |
| pmf0450 | Ginsenoside Rg1 | Terpene | -3.98 | down |
| pmf0467 | Morroniside | Terpene | -2.70 | down |
| pmf0486 | Bilobalide | Terpene | -3.88 | down |
| pmf0487 | Ginsenoside Ro | Terpene | 2.18 | up |
| pmf0345 | (-)-Epiafzelechin | Polyphenol | -13.8 | down |
| pme1562 | Epicatechin gallate | Polyphenol | -6.03 | down |
| pma0170 | N', N''-Diferuloylspermidine | Phenolamides | -2.59 | down |
| pma0692 | N', N''-di-p-coumaroylspermidine | Phenolamides | -2.06 | down |
| pmf0010 | Xanthotoxol | Phenylpropanoids | -19.4 | down |
| pmf0094 | Methyl p-coumarate | Phenylpropanoids | -18.8 | down |
| pmb0235 | O-Feruloyl coumarin | Phenylpropanoids | -2.23 | down |
| pmb1178 | O-Feruloyl 2-hydroxylcoumarin | Phenylpropanoids | -3.78 | down |
| pme3245 | Medicarpin | Phenylpropanoids | -2.56 | down |
| pme0387 | Homovanillic acid | Phenylpropanoids | 5.69 | up |
| pme0113 | γ-Glu-Cys | Amino acid and derivatives | -2.32 | down |
| pme0394 | 2-Methoxybenzoic acid | Organic acids and derivatives | -5.37 | down |
| pme0265 | Sebacate | Organic acids and derivatives | -3.12 | down |
| pme2603 | 3-Hydroxyanthranilic acid | Organic acids and derivatives | -3.08 | down |
| pmb3072 | 3-O-p-coumaroyl shikimic acid O-hexoside | Organic acids and derivatives | -2.46 | down |
| pmf0132 | Coumalic acid | Organic acids and derivatives | 16.0 | down |
| pme0291 | Dl-2-Aminooctanoic acid | Organic acids and derivatives | 2.56 | down |

Note: The metabolites listed in the table were fold change (Log_2_FC)>2.


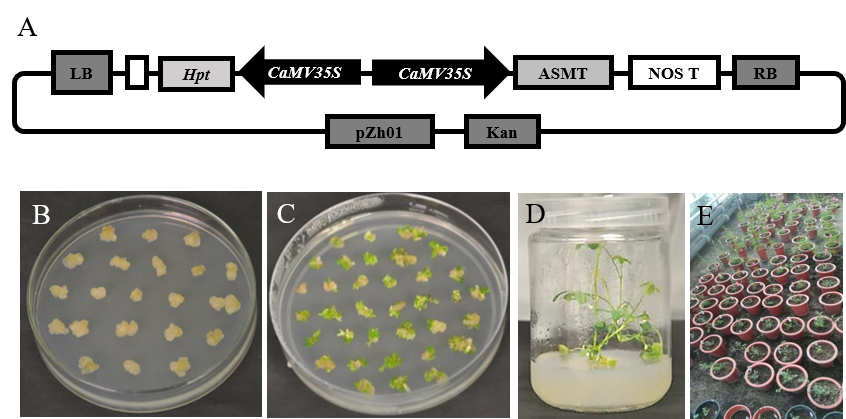
Fig. S1 Generation of *OE-MsASMT1* transgenic alfalfa

A, Schematic diagram of the T-DNA region of pZh01*-MsASMT1*; B, Callus induction; C, Somatic embryos induction; D, Resistant alfalfa plantlets rooting on MS medium; E, Resistant regeneration plantlets in pots.


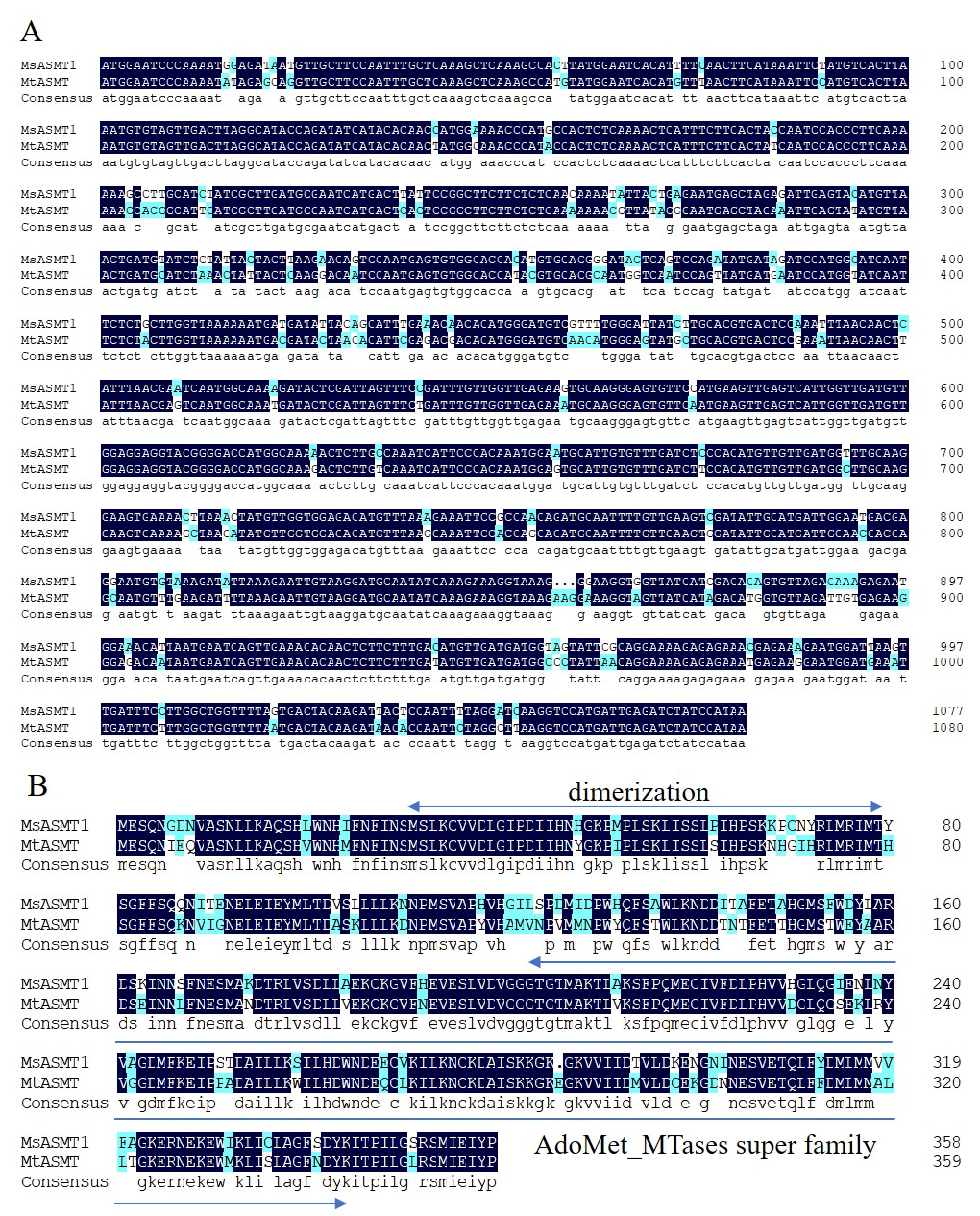
Fig. S2 Nucleotide (A) and amino acid (B) sequences alignment between MsASMT1 and MtASMT


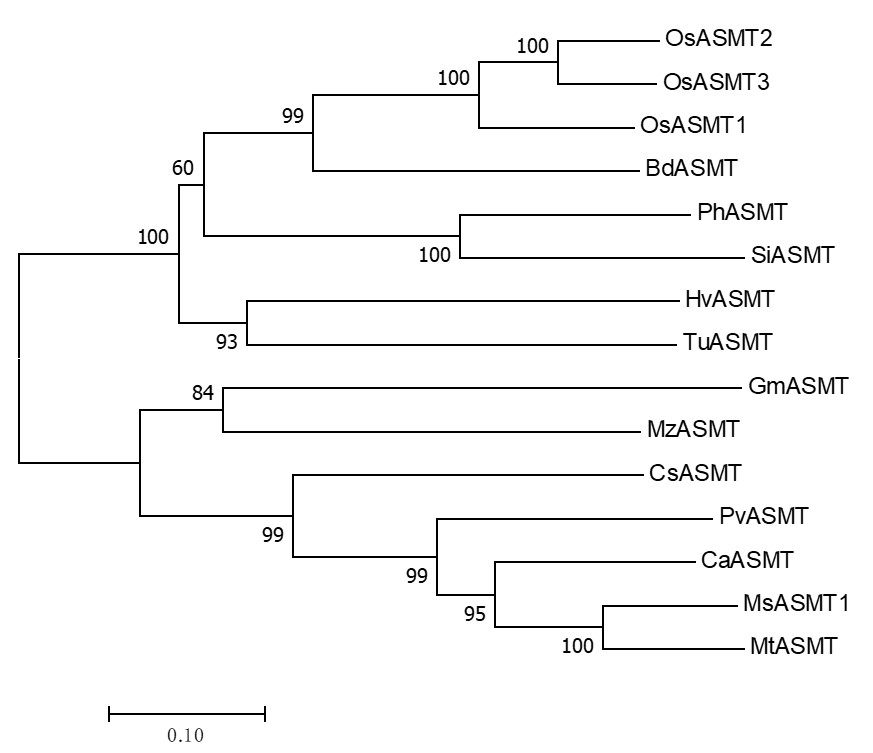
Fig. S3 Neighbor-joining phylogenetic tree of ASMT derived from various plant species

Note: *Oryza sativa* *OsASMT1* (AK072740), *OsASMT2* (AK069308), *OsASMT3* (AAL34945.1), *Malus zumi* *MzASMT* (KJ123721), *Phaseolus vulgaris* *PvASMT* (XP_007142077), *Setaria italica* *SiASMT* (XP_004973630.1), *Triticum urartu* *TuASMT* (EMS45259.1), *Brachypodium distachyon* *BdASMT* (XP_003571634.3), *Cicer arietinum* *CaASMT* (XM_004490639.2), *Cucumis sativus* *CsASMT* (XP_004151735), *Glycine max* *GmASMT* (XP_003536188), *Hordeum vulgare* *HvASMT* (BAK00281) and *Panicum hallii* *PhASMT* (XP_025822442.1).


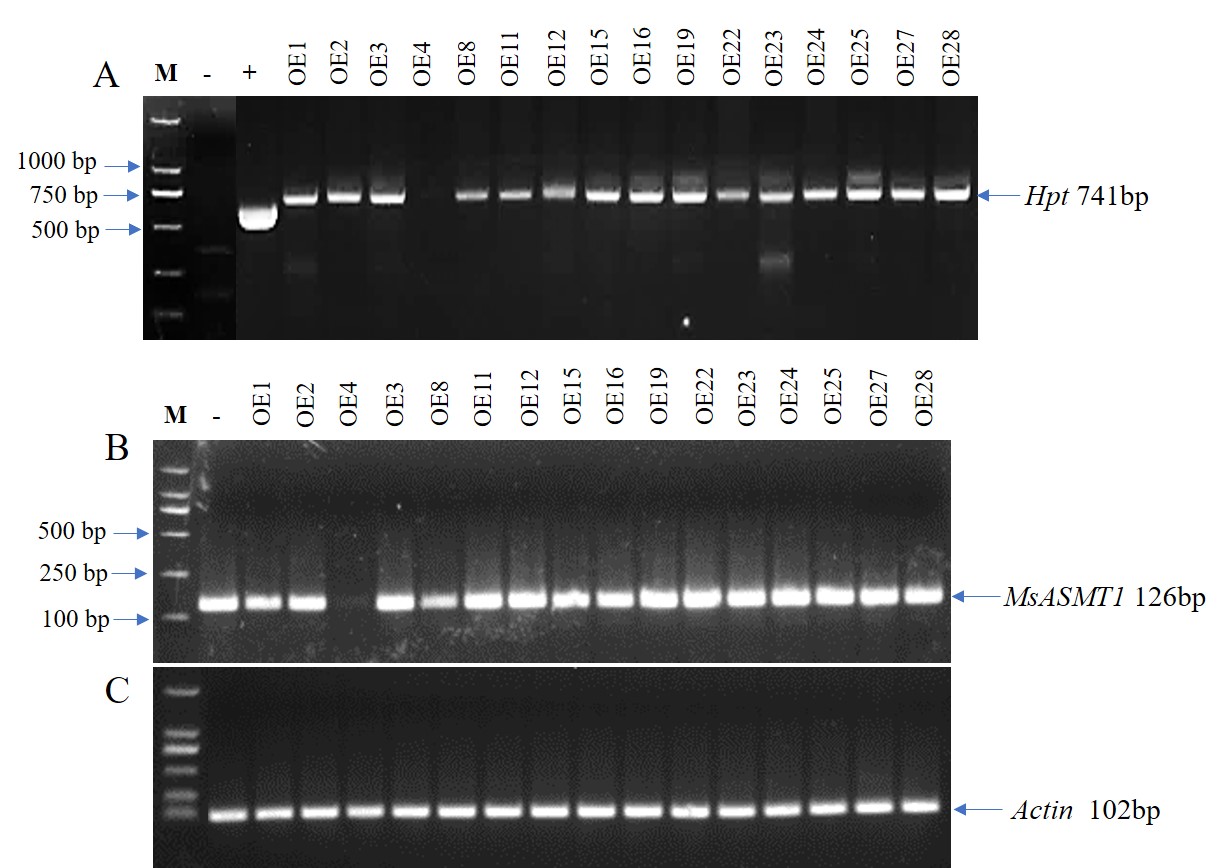
Fig. S4 PCR and RT-PCR analyses of *OE-MsASMT1* transgenic plants

A, PCR tests of partial *OE-MsASMT1* transgenic plants with the selectable marker gene *hpt*, M, marker D2000; -, negative control, the DNA of WT plant was used as a template; +, positive control, plasmid pZH01*-MsASMT1* was used as a template; OE1-28, the DNA of transgenic plants was used as templates; B-C, RT-PCR test of the specific *MsASMT1* gene in *OE-MsASMT1* transgenic plants (B), an alfalfa internal reference gene *actin* served as a template loading control (C), M, marker D2000; -, negative control, the cDNA of WT plant was used as a template; OE1-28, the cDNA of transgenic plants was used as templates.


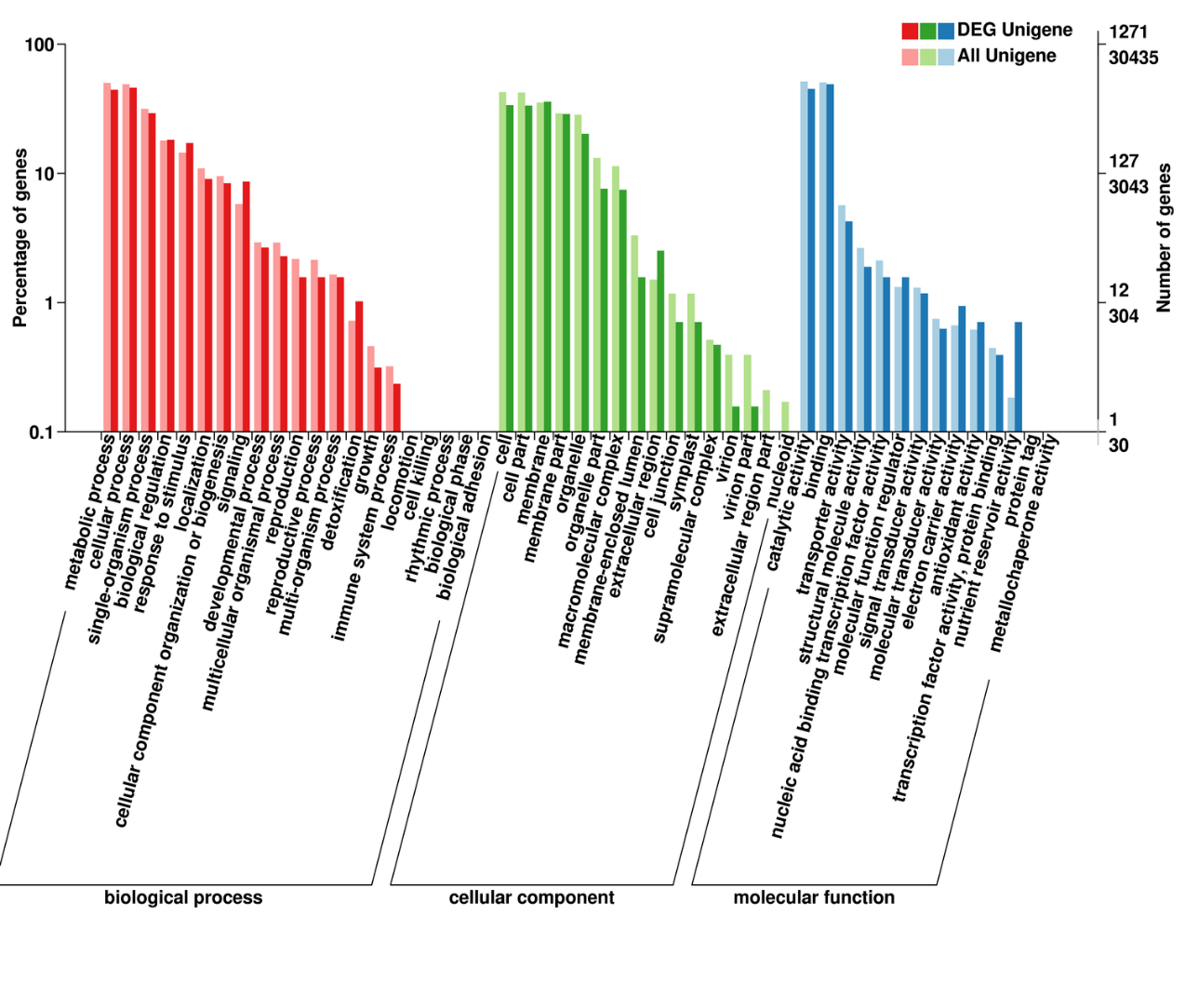
Fig. S5 GO classifications of DEGs between WT plants and *OE-MsASMT1* transgenic plants

Note: GO terms were functionally classified into three GO categories: biological process, cellular component and molecular function. The X-axis represents GO categories, Y-axis represents percentage of genes and number of genes respectively in each category.
